# Relationship Between Physical Activity and Autonomic Responses in Adults with Type 2 Diabetes

**DOI:** 10.3390/ijerph22111702

**Published:** 2025-11-11

**Authors:** Michela Persiani, Alessandra Laffi, Alessandro Piras, Andrea Meoni, Lucia Brodosi, Alba Nicastri, Maria Letizia Petroni, Milena Raffi

**Affiliations:** 1 Department of Biomedical and Neuromotor Sciences DIBINEM, Bologna University, 40126 Bologna, Italy; michela.persiani3@unibo.it (M.P.); alessandra.laffi3@unibo.it (A.L.); andrea.meoni@unibo.it (A.M.); 2 Department of Quality of Life Studies QUVI, Bologna University, 40126 Bologna, Italy; alessandro.piras3@unibo.it; 3 Clinical Nutrition and Metabolism Unit, IRCCS AOUBO, 40138 Bologna, Italy; lucia.brodosi2@unibo.it; 4 Department of Medical and Surgical Sciences, Bologna University, 40138 Bologna, Italy; alba.nicastri@studio.unibo.it (A.N.); marialetizia.petroni@unibo.it (M.L.P.)

**Keywords:** type 2 diabetes, heart rate variability, HRV, autonomic nervous system, physical activity, life style

## Abstract

Background: Cardiac autonomic dysfunction is a frequent complication of diabetes type 2 (T2DM). Heart rate variability (HRV) is a sensitive biomarker, but its relationship with habitual physical activity adjusted for metabolic and anthropometric factors remains underexplored. This study aimed to compare HRV indices between physically active and inactive adults with T2DM and assess the association between physical activity and clinical variables. Methods: In this cross-sectional observational study, 41 T2DM adults were classified as physically active (*n* = 22) or inactive (*n* = 19) using the short form of the International Physical Activity Questionnaire IPAQ-S. Resting HRV recordings were performed under standardized procedures. We analyzed the following time- and frequency-domain HRV indices: root mean square of successive heartbeat interval differences (RMSSD), standard deviation of normal-to-normal R-R intervals (SDNN), low-frequency (LF) and high-frequency (HF) power and their ratio (LF/HF). The analysis has been performed between-groups, and backward stepwise quantile regression examined the independent association of physical activity with HRV, adjusting for covariates. Results: Active participants exhibited higher HRV indices (SDNN *p* = 0.021; RMSSD *p* = 0.028; LF *p* = 0.032; HF *p* = 0.030), despite similar anthropometric and metabolic profiles. BMI correlated negatively with mean RR (ρ = −0.339, *p* = 0.030) and positively with mean HR (ρ = 0.339, *p* = 0.030). Physical activity was positively associated with LF (*p* = 0.015), and remained independently associated with SDNN (*p* = 0.021) and RMSSD (*p* = 0.048) after adjusting for HbA1c. Conclusions: Habitual physical activity was independently associated with enhanced autonomic modulation, with SDNN emerging as an early marker, supporting HRV as a biomarker for guiding exercise interventions in T2DM.

## 1. Introduction

Diabetes mellitus comprises metabolic disorders characterized by chronic hyperglycemia due to defects in insulin secretion or action [1]. Type 2 diabetes mellitus (T2DM) has emerged as one of the foremost global public health challenges of the twenty-first century, with the age-standardized rate of mortality reported to have increased by 13% from 2000 to 2019 in low- and middle-income countries [1]. In 2024, the International Diabetes Federation (IDF) estimates that 589 million adults aged 20–79 years are living with diabetes, and projects this number to exceed 850 million by 2050 [2]. Asia is a major area of the rapidly emerging T2DM global epidemic, with China (148 million cases) and India (212 million cases) represent together almost one-third of the global total, while age-standardized prevalence exceeds 25% in several Pacific Island states (e.g., Pakistan 30.8%, French Polynesia 25.2%, Kuwait 24.9%) [2,3,4]. In the European Region, where over 60 million adults live with diabetes, T2DM has an average prevalence of 8–9%, ranging from under 6% in some northern countries to above 12% in parts of southern Europe and the Mediterranean [1]. Accelerating urbanization, population ageing, sedentary behavior, and rising obesity are recognized as the key drivers of this epidemic [5].

Prolonged elevations in blood glucose (postprandial > 180 mg/dL; fasting ≥ 126 mg/dL) mark an early manifestation of diabetes [6] and, if left untreated, lead to widespread tissue injury [7]. Uncontrolled hyperglycemia drives both microvascular complications (retinopathy, nephropathy, and neuropathy) and macrovascular disease, including atherosclerotic cardiovascular events, stroke, and diabetic foot syndrome. Additionally, cardiac autonomic dysfunction in T2DM is frequently subclinical in its early stages and involves abnormalities in both parasympathetic and sympathetic control. According to a recent meta-analysis, cardiac autonomic neuropathy is associated with approximately a three-fold higher risk of cardiovascular events and all-cause mortality in individuals with diabetes [8,9]. 

Cardiac autonomic modulation, reflecting the balance between sympathetic and parasympathetic activity, is a critical indicator of cardiovascular health in T2DM [10]. It can be noninvasively assessed through heart-rate variability (HRV) indices: time-domain measures such as SDNN and RMSSD and high-frequency (HF) power reflect parasympathetic (vagal) outflow, while low-frequency (LF) power represents combined sympathetic and parasympathetic influences [11]. The HRV analysis can provide detailed information about the cardiac regulatory system, and it has been demonstrated that T2DM patients exhibit a strong decrease in HRV [11]. Alterations in HRV are common in diabetes and correlate with disease severity [12,13], and diabetic autonomic dysfunction has been linked to increased arterial stiffness, independent of age and blood pressure—thereby compounding cardiovascular risk [13,14]. Moreover, autonomic neuropathy in T2DM predicts adverse outcomes such as silent ischemia, arrhythmias, and sudden cardiac death, thus underscoring the clinical importance of early HRV assessment. 

The non-pharmacological management of T2DM hinges on comprehensive lifestyle modification, principally exercise, dietary adjustment, and behavioral counseling, and is supplemented by pharmacotherapy [1]. The World Health Organization (WHO) defines physical activity (PA) as any skeletal-muscle movement expending energy [15]. Clinical guidelines recommend that adults with type 2 diabetes engage in at least 150 min per week of moderate-to-vigorous aerobic physical activity, spread over a minimum of 3 days with no more than 2 consecutive inactive days, or alternatively 75 min of vigorous-intensity activity weekly. In addition, resistance training targeting major muscle groups should be performed 2–3 times per week, along with breaking up prolonged sitting every 30 min with light activity to improve glucose regulation [16]. Flexibility and balance exercises are also encouraged, particularly for older adults to reduce fall risk. These combined components form the foundation of contemporary exercise prescriptions in diabetes management [1,16]. Integrating these recommendations into routine care remains a major challenge, as most adults with T2DM do not achieve sufficient levels of physical activity. Breaking up prolonged sitting every 30 min with light activity (e.g., standing or short walks) further aids glucose regulation, while two to three sessions per week of flexibility and balance exercises (including yoga or tai chi) support mobility and fall prevention, especially in older adults [17]. Tailoring frequency, intensity, time, and type of exercise to each individual’s health status and preferences ensures maximal benefit and adherence, making a well-rounded activity program the cornerstone of effective diabetes management [18]. Regular physical activity is the cornerstone of this approach, as it improves insulin sensitivity and glucose tolerance, leading to reductions in glycated haemoglobin (HbA1c), triglycerides, blood pressure, and fat mass, while preserving lean body mass and increasing muscular strength [15]. Indeed, exercise training protocols have consistently demonstrated improvements in cardiorespiratory fitness and body composition among adults with T2DM [19]. Moreover, physical activity confers substantial cardiovascular benefits, mitigating risk factors such as hypertension and dyslipidemia, and is associated with lower all-cause mortality in T2DM populations [20,21]. Despite these benefits, most adults with T2DM fail to meet recommended physical activity levels [22,23]. 

To our knowledge, no study in T2DM has directly compared resting HRV profiles between habitual active and inactive patients while evaluating HRV in the context of key metabolic control variables (e.g., HbA1C, lipid profile) [11] and simultaneously adjusting for these parameters. Based on this, the present study aims to compare standard time- and frequency-domain HRV parameters in physically active versus inactive adults with T2DM, and to explore associations between HRV metrics and key clinical outcomes (HbA1c, triglycerides, cholesterol, and waistline). These insights aim to inform personalized exercise prescriptions and support the early identification of autonomic dysfunction in the clinical management of T2DM. 

## 2. Materials and Methods

### 2.1. Participants

A total of 41 T2DM subjects were recruited in this study (13 M and 28 F) with a mean age of 61.85 ± 10.55; all had been diagnosed for at least one year.

The participants were enrolled through different diabetic patients’ associations. Exclusion criteria were (i) patients with type 1 diabetes, or gestational diabetes; (ii) alcohol abuse or the use of recreational drugs that, in the opinion of the investigators, could compromise patient safety or interfere with study procedures; (iii) patients with clinically significant arrhythmias; (iv) known disorders affecting autonomic nervous system function; (v) patients with other diabetes-related complications including nephropathy, retinopathy, or peripheral neuropathy. This cross-sectional observational study, study was conducted in accordance with the Declaration of Helsinki, and approved by the Bioethics Committee of the University of Bologna, Italy, for studies involving human subjects (protocol n. 0283851, date of approval 4 November 2021). Patients consented to share their clinical data, collected at regular time points.

### 2.2. Procedure

#### 2.2.1. International Physical Activity Questionnaire (IPAQ) Questionnaire

Physical activity was assessed using the short form of the self-administered IPAQ [24] to classify participants into “physically active” and “inactive” groups. The IPAQ-S quantifies habitual physical activity over the preceding seven-day period across three intensity domains (vigorous activity, moderate activity, and walking), within all settings (leisure, domestic, work, and transport). Weekly duration (minutes) and frequency (days) in each domain are multiplied by standard metabolic equivalent (MET) values (8.0 for vigorous, 4.0 for moderate, 3.3 for walking), and these products are summed to yield a total physical activity score expressed in MET-minutes per week [25]. Participants reporting ≥700 MET-minutes/week were classified as “physically active,” while those below this threshold were designated “inactive.” This IPAQ-S–derived categorization served as the primary factor for group comparisons of HRV parameters and associated metabolic outcomes [24,25].

#### 2.2.2. Anthropometric Assessment

Participants underwent anthropometric measurements. Weight and height were recorded to calculate BMI (kg/m^2^). Waistline was measured at the midpoint between the lower rib margin and the iliac crest. All measurements were taken twice by the same operator, and the mean values were used for analysis.

#### 2.2.3. HRV Assessment

In the supine position, participants underwent a 10-min period of continuous, noninvasive beat-to-beat blood pressure monitoring (Portapres Model 2, 100 Hz; Amsterdam, The Netherlands) in a quiet, temperature—(22 °C) and humidity-controlled (52%) room [26]. Throughout this interval, subjects remained motionless and silent, breathing at 12–15 breaths per minute under metronome guidance (0.20–0.25 Hz). From the recorded pressure waveform, R–R intervals (Mean RR), resting heart rate (HR resting), systolic (fiSYS), diastolic (fiDIA), and mean arterial pressure (fiMAP) time series were extracted, visually inspected, and digitally filtered to remove artifacts. Heart rate variability (HRV) was analyzed in both the time and frequency domains using Kubios HRV v.2.0 (University of Kuopio, Kuopio, Finland). Time-domain indices included the root mean square of successive R–R differences (RMSSD), as a marker of short-term, vagally mediated variability, and the standard deviation of all normal-to-normal R–R intervals (SDNN), reflecting overall autonomic modulation. Spectral analysis via fast Fourier transforms yielded low-frequency (LF; 0.04–0.15 Hz) and high-frequency (HF; 0.15–0.40 Hz) components, corresponding to mixed sympathetic–parasympathetic and predominantly parasympathetic activity, respectively [10,27]. Both LF and HF are expressed in absolute power, calculated as ms^2^ per Hz (ms^2^/Hz) [27]. The LF/HF ratio was also computed as an index of sympathovagal balance under these controlled conditions [27].

### 2.3. Statistical Analysis

For the analysis of autonomic function, the last 5 min of recordings were used for calculations, as recommended by the guidelines for HRV analysis during short-term recording [10]. 

All continuous variables were first tested for normality using the Shapiro–Wilk test. 

Between-group comparisons (physically active vs. inactive) were performed using independent-samples *t*-tests for normally distributed variables (fiSYS, fiDIA, fiMAP, resting HR, Mean RR; Cohen’s d reported) and Mann–Whitney U tests for non-normally distributed variables (SDNN, RMSSD, LF, HF, LF/HF ratio, HbA1c, Triglycerides, Total Cholesterol, BMI, Waistline; effect size r reported). To assess associations between HRV parameters and anthropometric/metabolic variables (BMI, Waistline, HbA1c, Triglycerides and Total Cholesterol), Spearman’s rank correlation analyses were conducted (ρ and p-values reported). To determine whether group differences in HRV and hemodynamic parameters remained significant after adjusting for potential confounders, we fitted quantile regression models with robust variance estimation (backward stepwise selection, removal criterion *p* < 0.05), specifying each HRV or hemodynamic index as the dependent variable and including anthropometric/metabolic parameters and group as covariates. All statistical tests were two-tailed, and significance was set at *p*  <  0.05. Results are reported as mean ± SD for normally distributed continuous variables and median (interquartile range, IQR) for non-normally distributed continuous variables. Analyses were conducted using STATA SE 18.00 (StataCorp LP, College Station, TX, USA).

Due to the limited number of male participants in each physical activity category (physically active (PA): *n* = 5; physically inactive (PI): *n* = 8), a stratified gender analysis was not feasible without substantially reducing statistical power. Therefore, analyses were performed on the full group while adjusting for relevant covariates where possible.

## 3. Results

The sample consisted of 41 T2DM subjects classified as physically active (PA group *n* = 22) and physically inactive (PI group *n* = 19) according to the IPAQ self-guided survey [24]. The characteristics of patients are summarized in Table 1.

### 3.1. Anthropometric and Metabolic Parameters

The Mann–Whitney U test revealed no statistically significant differences between active and inactive participants in BMI, Waistline, HbA1c, Total Cholesterol, and Triglyceride levels. Although mean HbA1c was identical between groups, the active participants tended to exhibit lower mean values in other metabolic parameters compared to inactive ones (BMI: 30.33 ± 5.69 vs. 32.24 ± 5.88; Waistline: 103.41 ± 17.26 vs. 111.79 ± 15.01; Total Cholesterol: 166.72 ± 34.35 vs. 171.94 ± 38.31; Triglycerides: 122.05 ± 62.44 vs. 138.05 ± 60.19).

### 3.2. Resting Blood Pressure and Heart Rate

The independent T test revealed no statistically significant differences between active and inactive participants in resting blood pressure and heart rate. Data are reported using confidence interval (CI) and standard errors (SE). In all participants a significant negative correlation between Mean RR and BMI (Spearman’s ρ = −0.339, *p* = 0.030), and a significant positive association between Mean HR and BMI (Spearman’s ρ = 0.339, *p* = 0.030) was showed. Additionally, HbA1c was strongly positively associated with fiSYS (Spearman’s ρ = 0.501, *p* = 0.002). 

Quantile regression analysis confirmed a significant negative association between mean RR and BMI (β = −8.10, SE = 3.15, *p* = 0.014, 95% CI [−14.46, −1.73]) and a positive association between mean HR and BMI (β = 0.75, SE = 0.34, *p* = 0.033, 95% CI [0.06, 1.43]), while HbA1c was borderline positively associated with fiSYS (β = 0.55, SE = 0.27, *p* = 0.052, 95% CI [−0.01, 1.11]). No significant effect of physical activity group (active vs. inactive) was observed in any model, suggesting that, once HRV is “adjusted” for those covariates, activity level per se does not independently influence cardiac autonomic indices in adults with T2DM (Table 2).

### 3.3. Time-Domain HRV Indices

**Figure 1:**
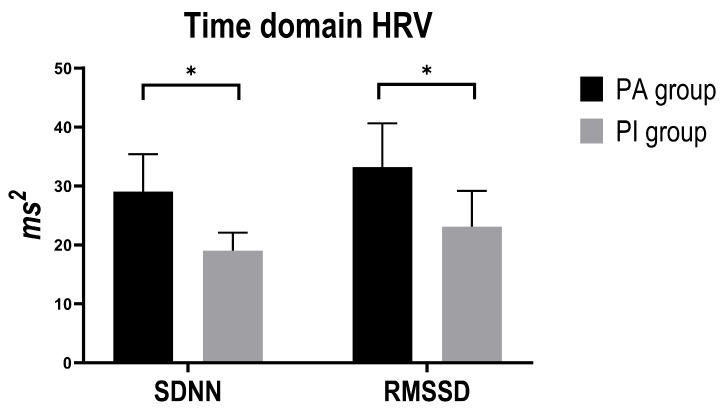
Histograms represent median and IQR of the Time Domain HRV parameters between PA group (black) and PI group (grey). Abbreviations: SDNN: standard deviation of normal-to-normal R-R intervals; RMSSD: square root of the mean squared differences of successive R-R intervals. Asterisks represent significant differences (*p* < 0.05).

The results of the Mann–Whitney tests showed that SDNN was higher in the active group (median 29.05 ms2, IQR 18.74–35.41) than in the inactive group (median 18.99 ms2, IQR 14.27–22.09); U = 121, z = 2.301, *p* = 0.021, effect size r = 0.36. Even RMSSD was greater in active participants (median 33.18 ms2, IQR 23.43–40.63) compared with inactive (median 23.10 ms2, IQR 19.85–29.19); U = 125, z = 2.196, *p* = 0.028, effect size r = 0.34 (Figure 1). 

Spearman correlations analysis revealed that both SDNN and RMSSD were positively associated with groups (Spearman’s SDNN: ρ = 0.364, *p* = 0.019; RMSDD: ρ = 0.347, *p* = 0.026) and HbA1c (Spearman’s SDNN: ρ = 0.389, *p* = 0.019; RMSSD: ρ = 0.350, *p* = 0.036). 

Quantile regression models adjusting for HbA1c showed that physical activity group (active vs. inactive) was significantly positively associated with both SDNN (β = 11.34, SE = 4.69, *p* = 0.021, 95% CI [1.79, 20.88]) and RMSSD (β = 11.12, SE = 5.41, *p* = 0.048, 95% CI [0.13, 22.12]), whereas HbA1c had no independent effect in either model. This suggests that, after accounting for glycemic control, being physically active is associated with enhanced cardiac autonomic modulation in adults with T2DM (Table 2).

### 3.4. Frequency-Domain HRV Indices

**Figure 2:**
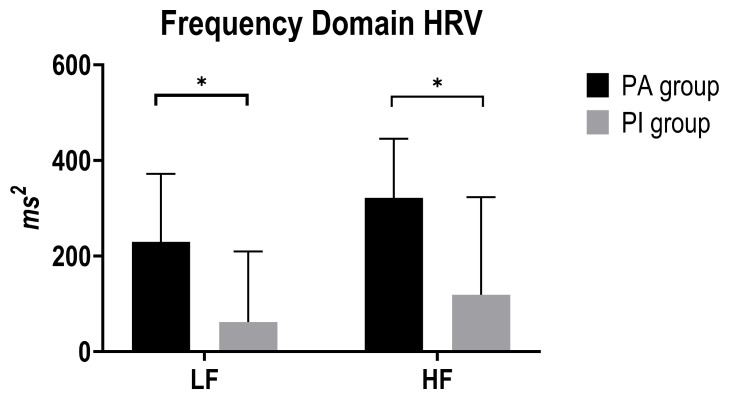
Histograms represent median and IQR of the Time frequency HRV parameters between PA group (black) and PI group (grey). Abbreviations: HF: high frequency; LF: low frequency. Asterisks represent significant differences (*p* < 0.05).

Mann–Whitney U tests revealed that LF power (ms^2^) was higher in the active group (median 229.83, IQR 53.58–371.89) versus inactive (median 61.66, IQR 27.55–209.66); U = 127, z = 2.144, *p* = 0.032, effect size r =0.34. Similarly, HF power (ms^2^) followed the same pattern: active median 321.82 (IQR 131.13–445.36) versus inactive 118.99 (IQR 80.00–323.11); U = 126, z = 2.170, *p* = 0.030, effect size r =0.34 (Figure 2). 

Spearman correlation analysis showed that both LF power (ms^2^) (Spearman’s ρ = 0.339, *p* = 0.030) and HF power (ms^2^) (Spearman’s ρ = 0.343, *p* = 0.028) were positively associated with physical activity levels. In addition, HF power (ms^2^) exhibited a positive association with HbA1c (Spearman’s ρ = 0.347, *p* = 0.038). The LF/HF ratio was negatively correlated with triglyceride levels (Spearman’s ρ = −0.373, *p* = 0.025). 

Quantile regression models revealed that the physical activity group was significantly and independently associated with LF power (ms^2^) (β = 190.04, SE = 74.51, *p* = 0.015, 95% CI [39.34, 340.75]). In contrast, neither HF power (ms^2^) nor the LF/HF ratio showed significant associations with activity group after adjusting for covariates, indicating that physical activity exerts its strongest independent influence on sympathetic modulation (LF power) among HRV components (Table 2).

## 4. Discussion

This study investigated the relationship between habitual physical activity, assessed via IPAQ, and cardiac autonomic function with key anthropometric/metabolic parameters in 41 adults with T2DM. Analyses compared unadjusted outcomes, then tested whether physical activity remained independently associated after adjusting for metabolic (HbA1c, lipids) and anthropometric (BMI, waistline) covariates.

HRV is increasingly recognized as a powerful predictor of cardiovascular risk, correlating not only with hyperglycemia and dyslipidemia but also with inflammation, genetic predisposition, and myocardial performance [28,29]. Due to its non-invasive nature, ease of use, and high sensitivity, HRV assessment has become a valuable tool for the early detection of diabetic autonomic neuropathy and related complications [30].

Although anthropometric, metabolic, and resting hemodynamic parameters were similar between physically active and inactive participants, unadjusted comparisons revealed significantly higher time-domain (SDNN, RMSSD) and frequency-domain (LF, HF) HRV indices in the active group, indicating enhanced cardiac autonomic modulation with greater overall variability and vagal tone. These findings underscore the contribution of habitual physical activity, assessed via the IPAQ survey, to autonomic regulation, even at activity levels below formal exercise thresholds [15,20]. Framed in physiological terms, SDNN captures global heart-rate variability and serves as the “gold standard” for cardiovascular risk stratification, especially in longer-term recordings, while RMSSD reflects beat-to-beat vagal activity with minimal respiratory or contextual confounding [27]. In the frequency domain, HF power predominantly indexes parasympathetic (vagal) modulation linked to respiration, LF power represents a mixture of sympathetic and parasympathetic influences (often baroreflex-mediated), and the LF/HF ratio provides a summary measure of sympathovagal balance [27].

Spearman’s rank correlations further underscored the intertwined relationships among activity, metabolic status, and autonomic function. Higher BMI was linked to both faster resting heart rates and shorter inter-beat intervals, while poorer glycemic control was associated with elevated systolic blood pressure. Turning to HRV, both time-domain (SDNN, RMSSD) and frequency-domain (LF, HF) indices rose in line with greater physical activity, and HF power also tracked with HbA1c levels. Conversely, the LF/HF ratio fell as triglyceride levels improved, pointing to a tighter sympathovagal balance in participants with healthier lipid profiles. These associations emerged despite similar BMI and glycemic control between groups, mirroring Picard et al. (2021), who demonstrated that even late-onset, modest increases in everyday activity can enhance HRV independent of changes in body composition [31]. 

Lastly, to determine whether group differences in HRV and hemodynamic parameters remain significant after adjusting for potential confounders, we fitted quantile regression models. Higher BMI was strongly and independently associated with impaired heart rate dynamics, showing a negative association with mean RR and a positive association with mean HR, whereas the effect of physical activity level (active, inactive) on these parameters becomes non-significant after adjusting for BMI. This finding suggests that, in this cohort of adults with T2DM, adiposity exerts a stronger influence on HRV than physical activity, consistent with previous findings that obesity is a major determinant of cardiac autonomic dysfunction, potentially overriding the benefits of physical activity [32]. Regarding hemodynamic parameters, HbA1c showed a borderline positive association with fiSYS, supporting the notion that hyperglycemia promotes vascular dysfunction and increases the risk of micro- and macrovascular complications [6]. 

Notably, physical activity defined by groups was independently and positively associated with SDNN and RMSSD, time-domain HRV indices reflecting global and parasympathetic cardiac modulation, even after adjusting for HbA1c. These findings suggest that, despite the strong adverse influence of glycemic control, being physically active may still confer beneficial effects on HRV, particularly on vagal-mediated components, in adults with T2DM [33,34]. Furthermore, physical activity defined by groups was also positively associated with LF power, a frequency-domain marker reflecting baroreflex-mediated autonomic modulation, with contributions from both parasympathetic and sympathetic activity [35], indicating a broader modulatory effect of exercise on autonomic balance [36]. Taken together, such autonomic changes contribute to improved cardiovascular regulation and reduced mortality risk in individuals with T2DM [31,37,38].

Overall, these findings seem to highlight the complex interplay between adiposity, glycemic control, and physical activity level in shaping cardiovascular autonomic and hemodynamic outcomes in adults with T2DM. While higher BMI and suboptimal glycemic control appear detrimental, regular physical activity remains an important modifiable factor that can partially mitigate autonomic dysfunction, particularly improving measures of parasympathetic modulation and overall HRV.

Nevertheless, our findings should be interpreted in light of certain limitations. The IPAQ reflects self-reported activity over a single week and may capture recent rather than habitual or long-term behavior. Moreover, its subjective nature may introduce recall or reporting bias. Because our study was cross-sectional, causal relationships cannot be inferred, and the durability of observed autonomic changes over time remains uncertain. Additionally, other potential confounders such as diabetes duration, medication use, and psychosocial stress were not assessed and could influence HRV. Another limitation is the gender imbalance in our sample, with women representing the majority of participants. Both cardiac autonomic responses and physical activity engagement can differ by gender, and physical activity is strongly shaped by sociocultural norms and perceived roles. Future research should recruit more balanced cohorts and explore potential gender-specific associations between habitual physical activity and autonomic modulation.

In conclusion, our findings suggest that habitual physical activity is independently associated with favorable autonomic modulation in adults with T2DM. These results reinforce the value of HRV as an early indicator of autonomic and cardiovascular health and highlight the potential benefits of even modest, unstructured physical activity. To translate these benefits into clinical practice, strategies to promote physical activity in this population should also address common psychological and physical barriers. Clinicians are encouraged to promote achievable and sustainable increases in daily movement to improve autonomic function and reduce long-term cardiovascular risk. Finally, future longitudinal studies are warranted to confirm these effects and to guide the development of targeted interventions.

## 5. Conclusions

In conclusion, our findings indicate that adults with T2DM who report higher habitual physical activity exhibit more favorable HRV indices, suggesting better autonomic modulation. While these associations support the importance of maintaining an active lifestyle, the cross-sectional design does not allow us to infer causal effects of physical activity on autonomic function. Longitudinal and intervention studies are needed to confirm whether increasing physical activity can directly improve HRV and to determine the dose and modalities that may yield the greatest benefits in this population.

## Figures and Tables

**Table 1:** Patient Characteristics.

	PA Group (*n. 22*)	PI Group (*n. 19*)
	***n.* (%)**	***n.* (%)**
**Gender**	M 5 (22.72)	M 8 (42.10)
**Anti-diabetic drugs**	14 (63.3)	9 (47.36)
**Insulin use**	5 (22.72)	2 (10.52)
	**Mean ± SD**	**Mean ± SD**
**Age (years)**	62.36 ± 11.32	61.26 ± 9.86
**Weight (Kg)**	81.11 ± 12.93	89.73 ± 20.51
**Height (m)**	1.64 ± 0.08	1.66 ± 0.11
**BMI (Kg/m^2^)**	30.32 ± 5.69	32.24 ± 5.88
**Waistline (cm)**	103.41 ± 17.26	111.79 ± 15.01
**HbA1c (mmol/mol)**	54.50 ± 9.96	54.38 ± 16.34
**FGP (mg/dl)**	130.94 ± 43.39	141.5 ± 54.20
**Total Cholesterol (mg/dl)**	166.72 ± 34.35	171.94 ± 38.32
**HDL (mg/dl)**	53.39 ± 13.69	56.33 ± 15.25
**LDL (mg/dl)**	90.89 ± 27.60	90.57 ± 32.51
**Triglycerides (mg/dl)**	122.06 ± 62.45	138.06 ± 60.19

Note. The table presents participant characteristics by group (PI = Physically Inactive; PA = Physically Active). Categorical variables (gender, use of antidiabetic drugs, and insulin) are expressed as *n.* and %, while continuous variables (age, anthropometrics, and clinical measures) are reported as mean ± SD. Abbreviations: BMI = Body Mass Index; FGP = Fasting Glucose Panel; HDL = High-Density Lipoprotein; LDL = Low-Density Lipoprotein.

**Table 2:** Quantile regression results for cardiovascular and HRV parameters (τ = 0.50).

Variables	Comparisons	Coefficient	Std. Error	t	P > |t|	95% CI Lower	95% CI Upper
**Mean RR**	PA vs. PI	−10.03	40.87	−0.25	0.80	−92.78	72.72
	BMI	−8.09	3.14	−2.57	0.014 **	−14.46	−1.73
**Mean HR**	PA vs. PI	0.87	3.98	0.22	0.83	−7.19	8.92
	BMI	0.75	0.39	2.22	0.033 *	0.06	1.43
**fiSYS**	PA vs. PI	8.27	6.35	1.30	0.202	−4.65	21.20
	HbA1c	0.55	0.27	2.02	0.052	−0.01	1.11
**SDNN**	PA vs. PI	11.34	4.69	2.42	0.021 *	1.79	20.88
	HbA1c	0.14	0.21	0.67	0.508	−028	0.56
**RMSSD**	PA vs. PI	11.12	5.41	2.06	0.048 *	0.13	22.12
	HbA1c	0.29	0.20	1.45	0.157	−0.12	0.71
**LF**	PA vs. PI	190.04	74.51	2.55	0.015 **	39.33	340.75
**HF**	PA vs. PI	167.63	94.57	1.77	0.086	−24.76	360.03
	HbA1c	5.16	4.80	1.08	0.29	−4.60	14.92
**LF/HF ratio**	PA vs. PI	0.13	0.19	0.65	0.518	−0.27	0.53
	Triglycerides	−0.00	0.00	−0.63	0.530	−0.00	0.00

Note. Values are regression coefficients (β) with standard errors in parentheses. CI = confidence interval. Model adjusted for metabolic covariates as indicated. τ = 0.50 (median regression). * *p* < 0.05; ** *p* < 0.01.

## Data Availability

The data that support the findings of this study are not openly available due to reasons of sensitivity but they are available from the corresponding author upon reasonable request. Data are located in controlled access data storage at the University of Bologna (Italy).

## Abbreviations

The following abbreviations are used in this manuscript:

BMI	Body Mass Index
CI	Confidence Interval
fiDIA	Diastolic Blood Pressure
FGP	Fasting Glucose Panel
HbA1c	Glycated Hemoglobin
HDL	High-Density Lipoprotein
HF	High-Frequency Power (HRV)
HR	Heart Rate
HRV	Heart Rate Variability
IQR	Interquartile Range
IPAQ	International Physical Activity Questionnaire
IPAQ-S	International Physical Activity Questionnaire, Short Form
LDL	Low-Density Lipoprotein
LF	Low-Frequency Power (HRV)
LF/HF ratio	Low-Frequency/High-Frequency Ratio
MAP/fiMAP	Mean Arterial Pressure
Mean RR	Mean of Normal-to-Normal RR Intervals
MET	Metabolic Equivalent of Task
PA	Physically Active
PI	Physically Inactive
RMSSD	Root Mean Square of Successive Differences (HRV index)
fiSYS	Systolic Blood Pressure
SD	Standard Deviation
SDNN	Standard Deviation of Normal-to-Normal RR Intervals (HRV index)
SE	Standard Error
T2DM	Type 2 Diabetes Mellitus
WHO	World Health Organization
